# Editorial: Vascular and valvular tissue engineering: Treating and modeling vasculopathies and valvulopathies

**DOI:** 10.3389/fcvm.2022.1092102

**Published:** 2022-11-30

**Authors:** Anthal I. P. M. Smits, Laura Iop, Adrian H. Chester

**Affiliations:** ^1^Department of Biomedical Engineering, Eindhoven University of Technology, Eindhoven, Netherlands; ^2^Institute for Complex Molecular Systems (ICMS), Eindhoven University of Technology, Eindhoven, Netherlands; ^3^Department of Cardiac, Thoracic, Vascular Sciences and Public Health, Padua Medical School, University of Padua, Padua, Italy; ^4^Heart Science Centre, Magdi Yacoub Institute, Harefield, United Kingdom; ^5^Imperial College London, National Heart and Lung Institute, London, United Kingdom

**Keywords:** tissue engineering and regenerative medicine, cardiovascular development, cardiovascular disease, biomaterials, heart valve replacement, biocompatibility, bioprosthetic heart valve, *in vitro* disease model

Considerable progress has been made in recent years with the development of tissue-engineered heart valves and blood vessels. As these laboratory-based projects make translational steps toward the clinic, a new set of hurdles need to be negotiated. The articles in this Research Topic address some of the most imminent question regarding cardiovascular tissue engineering: what inspiration can we draw from native valvular and vascular development? How is the integration and remodeling of a tissue-engineered graft influenced by immunological or hemodynamic conditions? Can we use tissue engineering methodologies to engineer *in vitro* and *ex vivo* disease models to systematically unravel pathophysiological processes and the result of interventions?

## Learning from and modeling biology

The main benefit of tissue-engineered substitutes is their intrinsic potential to grow and remodel in response to changing environmental conditions, analog to the native tissue that is being replaced. In-depth knowledge on the development and pathophysiological remodeling of native cardiovascular tissues is therefore indispensable. Starting from embryonic development, Henderson et al. provide an in-depth description on the state-of-the-art of (patho-)physiological development of the human arterial valves (i.e. semilunar; aortic and pulmonary valves). Importantly, they highlight that, while most of our knowledge regarding valve development is directly extrapolated from animal models, it is essential to also study these processes directly in humans; a challenge which is hampered by scarcity of donor material (Henderson et al.). When looking at postnatal valve remodeling, a quintessential characteristic is the capacity of the valves to remodel in response to changes in the hemodynamic loads. Van Hoof et al. review experimental and clinical data regarding pulmonary homograft remodeling after the Ross procedure, representing an extremely interesting study case, as it provides for a situation where a mature pulmonary valve is exposed to an extreme change in hemodynamic loads, being transplanted from the low pressure to the high pressure circulation.

Both these reviews highlight that there are still important knowledge gaps in our understanding of cardiovascular development and remodeling. Bioengineering and tissue engineering methodologies enable the development of *ex vivo* and *in vitro* tissue models to study cardiovascular remodeling and interventions in a mechanistic, well-controlled and potentially high-throughput manner, complementary to *in vivo* studies. In this context, Matos et al. describe the development of an *ex vivo* blood vessel culture setup in which vascular pathologies can be induced in a well-controlled biomimicking hydrodynamic environment. Chen et al. provide an overview of the literature with respect to bottom-up engineered *in vitro* models of atherosclerosis.

## Translatable tissue engineering methods: Natural matrix-based valve replacements

Despite considerable technical progress in cardiovascular tissue engineering, the translational challenges that have hampered clinical use of tissue-engineered cardiovascular substitutes so far, have become increasingly evident. Rizzi et al. provide an opinionated review on the various methodologies for heart valve tissue engineering, specifically focusing on broad translational potential. They advocate for the potential of *in situ* tissue engineering approaches, given their inherent reduced costs and logistical complexity when compared to *in vitro* cultured methods, and particularly, the use of natural scaffolds (e.g., decellularized allografts/xenografts) as the most native-like constructs available to date (Rizzi et al.).

One of the most important considerations for the use of decellularized valvular grafts is to minimize or harness the immunological response to the allogeneic or xenogeneic tissue, to prevent acute immunological rejection and adverse tissue remodeling (e.g. calcification, accelerated matrix degradation). Meng et al. describe the use of Sevelamer and an alternative cross-linking method to reduce the risk of calcification of bioprosthetic valves based on cross-linked bovine pericardium. Nevertheless, cross-linking inherently limits cellular ingrowth and functional matrix remodeling, and thereby abolishes the regenerative potential of bioprosthetic matrices. This has cued the development of matrix decellularization as an alternative treatment to create non-immunological matrices whilst maintaining regenerative potential. Oripov et al. report on a clinical study in which early binding of antibodies to decellularized allografts was longitudinally quantified in 20 patients with repaired congenital heart disease (median age 18 years). Their main finding is that there was increased antibody binding in some patients who received a decellularized aortic allograft, who subsequently developed valve degeneration within 28 days post-operatively (Oripov et al.). In addition, they observed interesting potential influences of patient age, sex, and patient-donor sex mismatch on antibody binding risk, although larger patient cohorts would be needed to draw robust conclusions on this (Oripov et al.).

As an alternative approach to using decellularized native tissues as source for bioprostheses, the use of decellularized *de novo in vitro* engineered tissues is being pursued to create natural-based cardiovascular replacements without the need for donor tissue. Poulis et al. provide an interesting perspective on the role of macrophages in the process of tissue remodeling and their reciprocal interactions with the extracellular matrix. This is building on the notion that, instead of avoiding the inflammatory response to the implanted graft, its potential can be harnessed to trigger and coordinate functional tissue regeneration. The importance of the extracellular matrix is further emphasized in the review by Sajeesh et al., who elaborate on the use of stem cell-secreted extracellular vesicles as a source of immunomodulatory and pro-regenerative factors to enhance functional matrix regeneration.

## Translatable tissue engineering methods: Using resorbable synthetic polymers for *in situ* tissue engineering

One of the downsides of natural-based cardiovascular grafts is the limited control over the structural and mechanical properties. Therefore, the use of resorbable synthetic polymers for regenerative cardiovascular grafts is being investigated, which offer relatively easy processing and a great level of control to engineer the ideal substitute. However, synthetic biomaterials lack the intrinsic bioactivity that is associated with the natural extracellular matrix. In an effort to combine the best of both worlds, Mudigonda et al. describe the development of a biohybrid scaffold material, consisting of decellularized pericardium coated with electrospun polycaprolactone-chitosan nanofibers to enhance mechanical strength and to improve cell homing.

One of the most critical challenges when using resorbable synthetic grafts for *in situ* tissue engineering is the balancing of resorption of the synthetic graft material with the formation of new tissue *in situ*, and to sustain function while the polymer is replaced by new tissue. Addressing this challenge, Tseng et al. report on the proof-of-concept for incorporation of carbon fibers into polycaprolactone heart valve scaffolds with the aim to sustain valve functionality while the polymeric mesh is replaced by new tissue. The balance between scaffold resorption and tissue formation is also addressed by Marzi et al., who report on the use of Raman microspectroscopy to characterize the extent and type of resorption of *in situ* tissue-engineered carotid artery grafts based on electrospun supramolecular elastomers implanted up to 12 months in sheep. Interestingly, they showed feasibility of measuring both the local scaffold degradation stage and the collagen maturation stage in the same location by obtaining the local molecular fingerprint using Raman microspectroscopy, thereby uniquely enabling the detailed and marker-free assessment of the local tissue-scaffold balance (Marzi et al.).

Taken together, the articles in this Research Collection address some of the most imminent current challenges pertaining to cardiovascular tissue engineering. Common denominators are the importance of cell-matrix interactions, inspired by the dynamism of native cardiovascular tissues, as well as immunological processes and the tailoring of biomaterials to modulate those. What is also apparent from this collection is that the scientific questions addressed within the tissue engineering field are increasingly converging with clinical and translational needs, rather than to be driven by technology push. These trends will accelerate translation of tissue engineering technologies and derivatives from bench to bed.

## Author contributions

AS prepared the initial draft. LI and AC reviewed and edited the manuscript. All authors contributed to the article and approved the submitted version.

## Conflict of interest

The authors declare that the research was conducted in the absence of any commercial or financial relationships that could be construed as a potential conflict of interest.

## Publisher's note

All claims expressed in this article are solely those of the authors and do not necessarily represent those of their affiliated organizations, or those of the publisher, the editors and the reviewers. Any product that may be evaluated in this article, or claim that may be made by its manufacturer, is not guaranteed or endorsed by the publisher.

